# Warming enabled upslope advance in western US forest fires

**DOI:** 10.1073/pnas.2009717118

**Published:** 2021-05-24

**Authors:** Mohammad Reza Alizadeh, John T. Abatzoglou, Charles H. Luce, Jan F. Adamowski, Arvin Farid, Mojtaba Sadegh

**Affiliations:** ^a^Department of Bioresource Engineering, McGill University, Montréal, QC H3A 0G4, Canada;; ^b^Management of Complex Systems Department, University of California, Merced, CA 95343;; ^c^United States Forest Service Aquatic Science Laboratory, Rocky Mountain Research Station, Boise, ID 83702;; ^d^Department of Civil Engineering, Boise State University, Boise, ID 83725

**Keywords:** wildfire, fire elevation, climate change, climate velocity, montane forests

## Abstract

Increases in burned area and large fire occurrence are widely documented over the western United States over the past half century. Here, we focus on the elevational distribution of forest fires in mountainous ecoregions of the western United States and show the largest increase rates in burned area above 2,500 m during 1984 to 2017. Furthermore, we show that high-elevation fires advanced upslope with a median cumulative change of 252 m (−107 to 656 m; 95% CI) in 34 y across studied ecoregions. We also document a strong interannual relationship between high-elevation fires and warm season vapor pressure deficit (VPD). The upslope advance of fires is consistent with observed warming reflected by a median upslope drift of VPD isolines of 295 m (59 to 704 m; 95% CI) during 1984 to 2017. These findings allow us to estimate that recent climate trends reduced the high-elevation flammability barrier and enabled fires in an additional 11% of western forests. Limited influences of fire management practices and longer fire-return intervals in these montane mesic systems suggest these changes are largely a byproduct of climate warming. Further weakening in the high-elevation flammability barrier with continued warming has the potential to transform montane fire regimes with numerous implications for ecosystems and watersheds.

## Significance

Forest fires of the western United States have advanced upslope over the past few decades, scorching territories previously too wet to burn. We document an upslope advancement of high-elevation fires of 7.6 m/y, a rate comparable to the elevational velocity of vapor pressure deficit of 8.9 m/y. Strong interannual links between aridity and high-elevation forest fires and reduced influence of fire exclusion policies in montane mesic forests imply such changes are a byproduct of climate warming. We estimate that increased aridity between 1984 and 2017 exposed an additional 81,500 km^2^ of western US montane forests to fires. These changes have significant implications for terrestrial carbon storage, snowpack, and water quantity and quality.

Fire is an integral component of most forested lands and provides significant ecological services (1). However, burned area, fire size, the number of large fires, and the length of fire season have increased in the western United States in recent decades (2, 3). Increasing fire activity and the expansion of wildland urban interface (4) collectively amplified direct and indirect fire-related loss of life and property (5, 6) and contributed to escalating fire suppression costs (7). While increased biomass due to a century of fire exclusion efforts is hypothesized to have partially contributed to this trend (8), climate change is also implicated in the rise of fire activity in the western United States (9–11).

Although increases in forest fire activity are evident in all major forested lands in the western United States (2, 12, 13), an abundance of moisture—due to snowpack persistence, cooler temperatures, and delayed summer soil and fuel drying—provides a strong buffer of fire activity (13) and longer fire-return intervals (14) at high elevations. Recent studies, however, point to changing fire characteristics across many ecoregions of the western United States (15), including high-elevation areas of the Sierra Nevada (16), Pacific Northwest, and Northern Rockies (12, 17). These studies complement documented changes in montane environments including amplified warming with elevation (18), widespread upward elevational shift in species (19), and increased productivity in energy-limited high-elevation regions that enhance fuel growth and connectivity (20). These changes have been accompanied by longer snow-free periods (21), increased evaporative demand (9), and regional declines in fire season precipitation frequency (11) across the western United States promoting increased fuel ignitability and flammability that have well-founded links to forest burned area. A warmer climate is also conducive to a higher number of convective storms and more frequent lightning strikes (22).

In this study, we explore changes in the elevational distribution of burned forest across the western United States and how changes in climate have affected the mesic barrier for high-elevation fire activity. We focus on changes in high-elevation forests that have endured fewer direct anthropogenic modifications compared to drier low-elevation forests that had frequent low-severity fires prior to European colonization and have been more subject to changes in settlement patterns as well as fire suppression and harvest (23, 24); we also pose the following questions: 1) Has the elevational distribution of fire in the western US forests systematically changed? and 2) What changes in biophysical factors have enabled such changes in high-elevation fire activity? We explore these questions across 15 mountainous ecoregions of the western United States using records from large fires (>405 ha) between 1984 and 2017 [Monitoring Trends in Burn Severity (MTBS) (25)], a 10-m–resolution digital elevation model, and daily high-spatial–resolution surface meteorological data [gridMET (26)].

We focus on the trends in Z_90_—defined as the 90th percentile of normalized annual elevational distribution of burned forest in each ecoregion. Here, the term “normalized” essentially refers to the fraction of forest area burned by elevation. We complement this analysis by examining trends in burned area by elevational bands and using quantile regression of normalized annual forest fire elevation. We then assess the interannual relationships between Z_90_ and vapor pressure deficit (VPD) and compare the upslope advance in montane fire to elevational climate velocity of VPD during 1984 to 2017. Specifically, we use VPD trends and VPD–high-elevation fire regression to estimate VPD-driven changes in Z_90_ and BA_90_— defined as annual burned area above the 90th percentile of forest elevational distribution in each ecoregion—during 1984 to 2017.

## Results

### Observed Changes in High-Elevation Forest Fires.

**Fig. 1.**
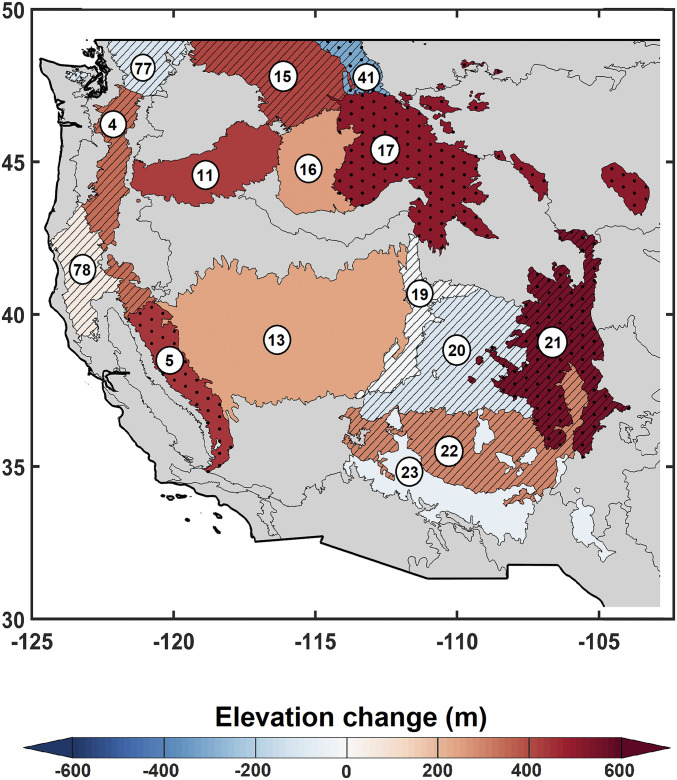
High-elevation fires are generally moving upslope across mountainous western United States. Changes in Z_90_ during 1984 to 2017 are presented. The dotted area represents statistically significant monotonic trend at the 5% level using the Mann–Kendall trend test. The hatched areas are associated with ecoregions with at least 10% length of record (4 y) excluded from the analysis due to absence of fire. The gray shaded ecoregions are not included in the analysis. The ecoregion names are as follows: 4: Cascades, 5: Sierra Nevada, 11: Blue Mountains, 13: Central Basin and Range, 15: Northern Rockies, 16: Idaho Batholith, 17: Middle Rockies, 19: Wasatch and Unita Mountains, 20: Colorado Plateaus, 21: Southern Rockies, 22: Arizona/New Mexico Plateau, 23: Arizona/New Mexico Mountains, 41: Canadian Rockies, 77: North Cascades, and 78: Klamath Mountains/California High North Coast Range.

We document an upslope advancement of Z_90_ by a median of 252 m (95% CI of −107 to 656 m) during 1984 to 2017 across the mountainous ecoregions of the western United States (Fig. 1 and *SI Appendix*, Table S1). Positive trends in Z_90_ were observed in 10 of 15 ecoregions, with the greatest upslope advance in the Southern Rockies, Middle Rockies, and Sierra Nevada ecoregions (550, 506, and 444 m during 1984 to 2017, respectively; all three are statistically significant at the 5% level; Fig. 1). Nonsignificant declines in Z_90_ were observed for a few ecoregions—a potential artifact of excluding years without fires that primarily (93%) occurred in the in the 1980s and ‘90s. Signal-to-noise problems (e.g., varied size of ecoregions, high interannual variability, and Z_90_ being a derivative of more widely used burned area) impede the detection of widespread statistically significant trends across ecoregions (4 of the 15 ecoregions had significant Z_90_ trends; Fig. 1).

**Fig. 2.**
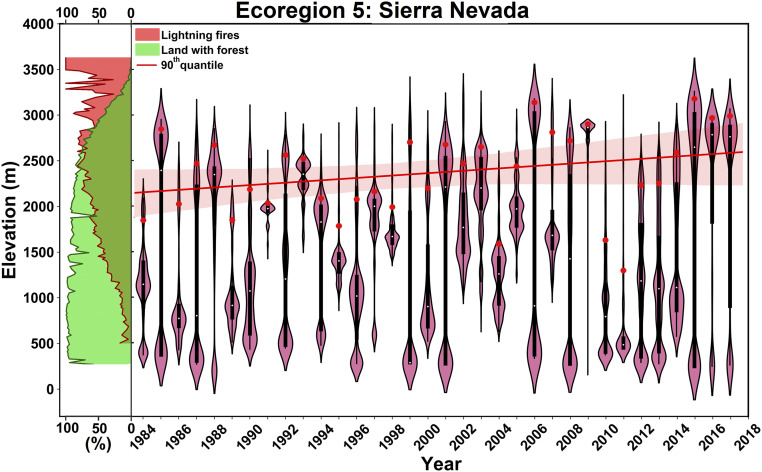
Normalized annual elevational distribution of burned forest for Sierra Nevada. Percent forest cover across the elevational band is shown with green shaded area, percent lightning-started fires are shown with red shaded area on the *Left*, and Z_90_ for each year is marked with red dots. The red line indicates the slope of Z_90_ during 1984 to 2017, with uncertainty range shown with shaded area.

The Sierra Nevada ecoregion showed a statistically significant 444 m increase in Z_90_ over the study period (Fig. 2; other ecoregions shown in *SI Appendix*, Fig. S1). Burned forest in the Sierra Nevada ecoregion above 3,000 m was rare during 1984 to 2000 (433 ha/y) but has become more common during 2001 to 2017 (4,130 ha/y; *SI Appendix*, Table S2). Forest burned area above the elevation of 3,000 m in Sierra Nevada, for example, accounted for 30% of normalized annual burned area in 2015 (Fig. 2). Furthermore, fires advanced upslope to the highest-elevated forested lands during 2001 to 2017, implying disappearance of the mesic barrier to fire in recent years (Fig. 2).

We complement our analysis of trends in Z_90_ by examining differences in forest burned area by elevational bands between 1984 to 2000 and 2001 to 2017 (*SI Appendix*, Fig. S2). The greatest median rates of increase (439 and 271%) in forest burned area among the 15 mountainous western US ecoregions occurred in the high-elevation bands of >3,000 m and 2,500 to 3,000 m, respectively (*SI Appendix*, Table S2). Lesser increases were observed for lower-elevation forested regions (e.g., 73% for the 1,000 to 1,500 m band; *SI Appendix*, Table S2). These findings are in accordance with previous regional studies (16, 17). Similarly, quantile regression of normalized annual elevational distribution of burned forest showed significantly greater upslope trends for higher-elevation areas compared to their lower-elevation counterparts (*SI Appendix*, Fig. S3 and Table S3).

The proportion of fires that were caused by lightning increased with elevation across ecoregions, with high-elevation fires being chiefly lightning caused (*SI Appendix*, Fig. S1). Notably, studies have shown that lightning-caused fires have been the major contributor to the increased burned area in the western United States (2, 27). We did not find evidence of significant linear correlations between the number of warm season (May to September) cloud-to-ground lightning strikes and any of the high-elevation fire metrics, suggesting that variability and changes in lightning occurrence are not directly implicated in the upslope advance of fires (*SI Appendix*, Table S4).

### Links between Climate Aridity and High-Elevation Forest Fires.

Z_90_ was distinctly higher during high-VPD warm seasons (VPD in the upper tercile) than low-VPD warm seasons (VPD in the lower tercile) (*SI Appendix*, Fig. S4). We also found positive interannual correlations between warm season VPD and Z_90_ for a majority of regions (*SI Appendix*, Table S5), reaching statistical significance (*P* < 0.05) for 6 of the 15 ecoregions. Analyses with warm season climatic water deficit produced similar results (*SI Appendix*, Table S5). Furthermore, we found strong positive correlations between the forest burned area above different elevation thresholds [>2,000 m, >2,500 m, and >90th percentile of forest elevational distribution in each ecoregion (i.e., BA_90_)] and aridity with significant correlations for 14 of the 15 ecoregions (*SI Appendix*, Table S6 for VPD and *SI Appendix*, Table S7 for climatic water deficit).

**Fig. 3.**
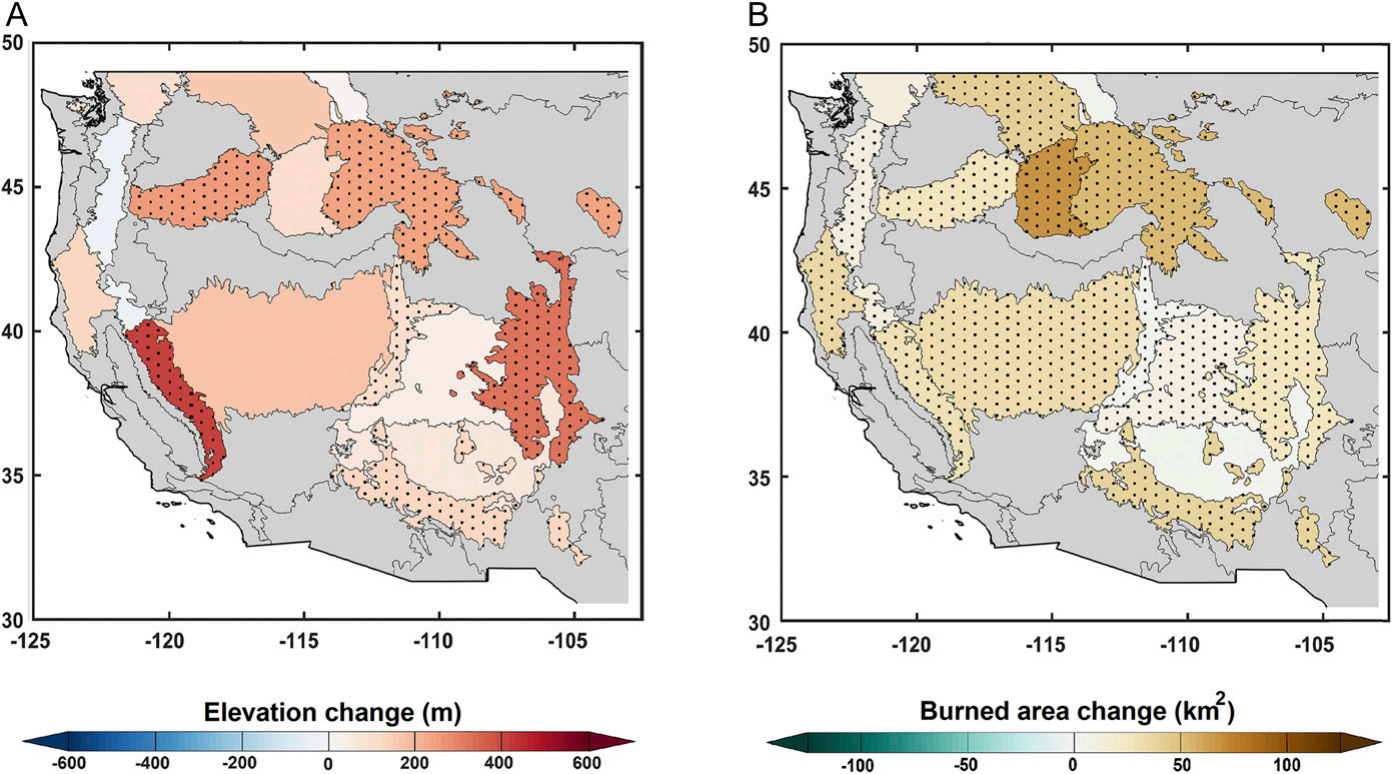
High-elevation forest fire activity increases in response to increasing VPD. (*A*) Change in Z_90_ and (*B*) change in annual BA_90_ as a result of increase in VPD from 1984 to 2017. The dotted area represents statistically significant linear correlation among warm season VPD and Z_90_ (*A*) and BA_90_ (*B*) at the 5% level.

Regression analysis indicates a distinct upslope advance in Z_90_ with increasing warm season VPD (*SI Appendix*, Table S8). We calculate VPD-driven trends in Z_90_ during 1984 to 2017 as the product of VPD trend and the VPD-Z_90_ regression. These results suggest a median upslope advance of 120 m in Z_90_ across the montane ecoregions of the western United States during 1984 to 2017 (Fig. 3*A*). Excluding years with no fire activity from the analysis degrades the VPD-Z_90_ sensitivity, and hence our estimate is likely conservative. A similar regression-based attribution applied to BA_90_ indicated that VPD-driven annual BA_90_ increased by 384.2 ${\text{km}}^{2}$ (256%) in the western United States during 1984 to 2017 (*SI Appendix*, Table S9). Individual ecoregions show a median increase in VPD-driven annual BA_90_ of 27.3 ${\text{km}}^{2}$ (190% increase) during 1984 to 2017 (Fig. 3*B* and *SI Appendix*, Table S9).

We also used a hypsometric approach that builds off the VPD-driven trends in Z_90_ to approximate the additional forested land susceptible to fire due to the waning of the high-elevation flammability barrier. We estimate an additional 11% (81,500 km^2^) of forested land has become vulnerable to fire across the western United States from 1984 to 2017. Individual ecoregions show various degrees of sensitivity with fraction of additional forested land vulnerable to fire ranging between −1 and 27% due to varied VPD-Z_90_ sensitivities and the hypsometry of forested land by ecoregion (*SI Appendix*, Table S8). These findings corroborate the upslope advance in fires presented in Fig. 1 and *SI Appendix*, Fig. S3.

**Fig. 4.**
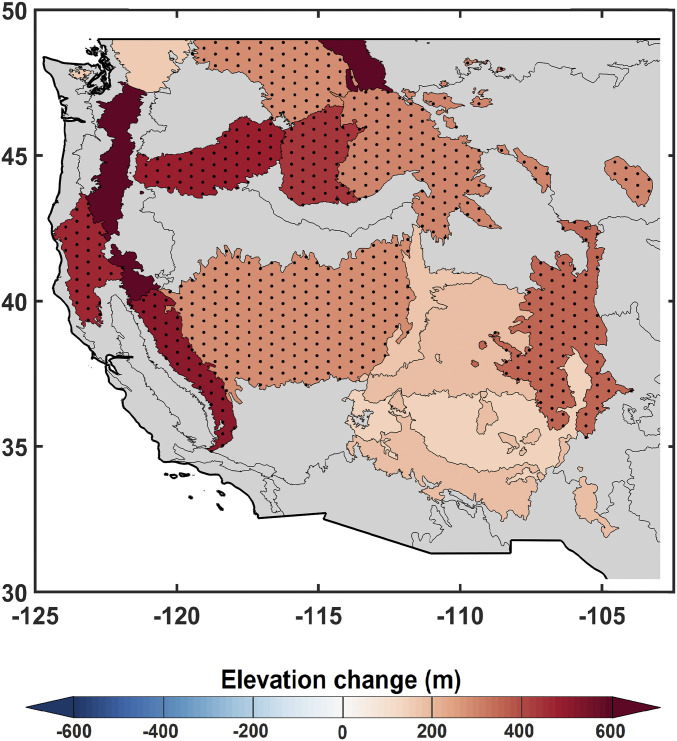
Elevation trends for warm season VPD isolines across the mountainous western United States during 1984 to 2017. The dotted areas represent statistically significant (5% level) monotonic trends (Mann–Kendall test).

Finally, we complement the observational evidence of upslope advance in Z_90_ by calculating vertical climate velocities of VPD. The median vertical advancement of VPD across ecoregions was 295 m (59 to 704 m; 95% CI) during 1984 to 2017 (Fig. 4 and *SI Appendix*, Table S1). Furthermore, a sensitivity analysis shows that ∼60% of the increase in VPD during 1984 to 2017 can be attributed to increased air temperature (*SI Appendix*, Fig. S5)—implicating warming in the upslope advance of fires. These results together with VPD-based estimates of Z_90_ provide complementary evidence that increased VPD with warming has weakened the mesic barrier for high-elevation fires, thereby enabling the documented shift in forest fire activity to higher elevation.

## Discussion

Fires in high-elevation forests are typically associated with return periods of one to multiple centuries (17). Recent regional studies have pointed to more frequent high-elevation forest fires in Sierra Nevada (16) and portions of the Pacific Northwest (17), but a large-scale and robust analysis of forest fire elevational changes has not been previously undertaken. Herein, through a comprehensive analysis of all large fires during 1984 to 2017 in mountainous ecoregions of the western United States, we show that high-elevation fires advanced upslope by 252 m (median across 15 ecoregions) during 1984 to 2017. We additionally found that the upslope advance was generally greater for higher-elevation fires than at lower elevations. Increased fire activity in subalpine forests is arguably more directly linked to climate warming compared to changes in drier low-elevation forests given the relatively reduced influence of fire exclusion and land management practices in remote high-elevation forests (17, 24). We show that the high-elevation mesic barrier to fires has advanced upslope—disappearing for some ecoregions in recent years—and fires are increasingly burning at higher elevations. Similarly, we find an upslope VPD advancement of 295 m during 1984 to 2017, corroborating the waning of high-elevation flammability barriers with a warming climate.

Increased high-elevation burned forest is associated with significant implications ranging from biodiversity loss and vegetation conversion to change of snowpack and landscape transformation. For example, high-elevation mountains are water towers of the world providing freshwater to millions of people, the contribution of which to annual basins’ water yield is disproportionally large due to climatic gradients at the high-elevation areas (28). By modifying vegetation cover and soil characteristics, fires modify hydrological balance of watersheds (29). Fires also impact snow accumulation, redistribution, and melt, which are particularly important at high elevations (30). In addition, severe fires can remove standing trees that are anchor points stabilizing snowpack, which together with heavier snowpack due to lower canopy interception enhance the frequency and magnitude of avalanches (31). Moreover, high-elevation fires reduce sediment retention capabilities of mountain stream networks and significantly reduce sediment residence time at high elevations (32). Diminished sediment retention capacity can induce substantial bedrock river incision and reduce active floodplain extent, which can in turn change river morphology and transform mountain landscapes (33).

Forest fires often occur during extended drought conditions with compounding impacts on the vulnerability of aquatic species to such increasingly frequent disturbances (34). Cold-water mountain streams are home to a variety of small-range endemic species (35) that may face extinction as their habitat shrinks or vanishes with stream flow temperatures increasing in response to escalating high-elevation air temperatures (18). Stream temperature increases within fire burned areas can be 2 to 3 times higher than that of the basin-wide average due to enhanced solar radiative forcing that reaches streams in the absence of canopy cover (36). As fires increasingly burn canopy at higher elevations, thermal habitat losses at coldest headwaters may become prevalent, causing extinction of some endemic species. Mountainous areas are climatically isolated with high resistance to species movement given their high climatic gradients; hence, increasing disturbances such as fires can have significant implications for the survival of endemic species (37), undermining the ability of montane and subalpine landscapes to provide refugia to many species as climate warms (38). Furthermore, since fire has been historically rare in high-elevation forests, increased fire occurrence can transform the structure and functioning of the montane regions, with cascading impacts for the landscape, lowland biomes, and human livelihood.

In this paper, we show fire activity is increasing disproportionally at high-elevation compared to low-elevation forests of the western United States. Here, we demonstrate changes in elevational distribution of fire activity in high-elevation forests, where fire has historically been quite rare and fire regimes have seen limited anthropogenic drivers such as land use and fire suppression. Compounding the direct climate-driven increases in high-elevation fires are large increases in burned area across the western United States. During years with widespread fire activity and strained fire suppression resources, we hypothesize the fire suppression activities are focused in mid- to low-elevation forests collocated with human settlement and infrastructure, further enabling high-elevation fires to burn with limited suppression. Climate warming is expected to continue increasing forest fire activity while fuel remains available (15, 39). This trend is also expected for high-elevation burned areas (40), which are believed to be more sensitive to the warming trend (17, 41). Our results also show that increasing productivity in high-elevation energy-limited forests also may have contributed to the growth of high-elevation fires in some ecoregions (*SI Appendix*, Tables S10 and S11), although flammability is posited to be the primary limiting factor in these mesic regions as supported in global climate–fire relationships (42) and paleofire analyses that show increased occurrence of high-elevation fires during warm epochs throughout the Holocene (43–45).

## Methods

Throughout this manuscript, we refer to “Z_90_” as the 90th percentile of normalized annual elevational distribution of burned forest in each ecoregion. Here, the term “normalized” essentially refers to the fraction of forest area burned by elevation. Furthermore, we refer to “BA_90_” as the total annual forest burned area above the 90th percentile of the forest elevational distribution in each ecoregion.

We focus on the forested areas of the mountainous western United States in this study, defined as grid cells with forest or woodland classification according to the Environmental Site Potential dataset of the Landfire Program (https://www.landfire.gov/esp.php). Analyses are conducted on fires larger than 405 ha—obtained from the MTBS program (MTBS.gov)—separately for level III Omernik ecoregions (46) (https://www.epa.gov/eco-research/ecoregions). All 30-m burned grids, except for those marked as “unburned to low-severity,” that coincide with forested lands are included in this study (binary analysis: burned or unburned). Source of ignition is determined from the Fire Program Analysis-Fire Occurrence Database [FPA-FOD (47)] (https://www.fs.usda.gov/rds/archive/catalog/RDS-2013-0009.4). MTBS data covers 1984 to 2017 and FPA-FOD covers 1992 to 2015. Digital Elevation Model (DEM) at 10-m (1/3 arc-second) horizontal resolution is obtained from the National Elevation Dataset (https://ned.usgs.gov) and used to determine the elevation of burned forest. For the analysis of interannual relationships among fire and normalized difference vegetation index (NDVI) and lightning strikes, we use advanced very high resolution radiometer (AVHRR)-based NDVI data at ∼5-km horizontal resolution during 1984 to 2017 (https://www.ncdc.noaa.gov/cdr/terrestrial/normalized-difference-vegetation-index) and cloud-to-ground lightning data from two sources: National Lightning Detection Network from 1990 to 2009 and North American Precision Lightning Network from 2010 to 2017. We removed low-intensity positive strikes to account for temporal inhomogeneities in the record as in Abatzoglou et al. (48).

We determine the elevational distribution of burned forest by overlaying grids of burned area, forest cover, and a DEM. We define two variables at each forested grid cell for a particular year: whether or not it has burned, B, taking on discrete values of 1 and 0, and elevation, E, a nominally continuous variable. One could estimate the unconditional probability of a grid cell burning, P(B), as the fraction of the forested area of each ecoregion that burned in a year. Our interest is in where in the elevation fires burn, so we compute the probability that a grid cell burns conditioned on elevation, P(B|E), which could be conceptualized as the cumulative probability of burn as one increments across the elevations in a given ecoregion. We are testing the hypothesis that a disproportionate change in burned areas has occurred across elevation in the past decades (i.e., a shift has materialized in the relative likelihood of fire at higher elevations compared to lower elevations in an ecoregion). To measure this, we normalize the conditional probability by the unconditional probability to generate $\frac{P\left(B\text{|}E\right)}{P\left(B\right)}$.

One could calculate the conditional probability P(B|E) using a series of elevation bands and aggregating the occurrence of the discrete variable B across bands. Bayes theorem, however, offers a more robust calculation—as it does not depend on the analysis choices used for elevation bands—by reframing the calculation in terms of the variable E:

$$P\left(B|E\right)=\frac{P\left(E|B\right)P\left(B\right)}{P\left(E\right)}\;,$$ [1]

or even more directly for the normalized variable of interest,

$$\frac{P\left(B|E\right)}{P\left(B\right)}=\frac{P\left(E|B\right)}{P\left(E\right)},$$ [2]

where P(E) is the empirical probability function of forest elevation, and P(E|B) is the empirical probability function of the elevation of forest burned cells. In Fig. 2 and *SI Appendix*, Fig. S1, we plot the elevational derivative of the resultant cumulative probability function, $\frac{d}{dE}\left[\frac{P\left(B\text{|}E\right)}{P\left(B\right)}\right]$—in other words, density: $\frac{p\left(B\text{|}E\right)}{p\left(B\right)}$—to show where in the elevation fire is most prevalent in a given year.

Quantile regression is then performed on the normalized annual elevational distribution of burned forest. Years without large forest fires are excluded from the analysis. The slope of the 90th quantile (meters per year) is multiplied by the number of years with forest fire activity to calculate the increase in Z_90_ for each ecoregion during 1984 to 2017. Similarly, the lower and upper bounds of the 95% CI for the slope of the 90th quantile (meters per year) are multiplied by the number of years with forest fire activity to estimate the 95% CI of Z_90_ trends for each ecoregion. Median of the lower and upper bounds of the uncertainty range across studied ecoregions are reported as the median 95% CI range in this paper (*SI Appendix*, Table S1).

For the fire–climate relationship and climate velocity analyses, we obtain daily VPD from gridMET (26) (www.climatologylab.org/gridmet.html). Because VPD incorporates both the effects of soil wetting variations as well as the radiative and advective aspects of the energy balance, it efficiently combines their effects, and VPD typically has a higher correlation with forest fire extent than other measures (11, 49). We also analyze correlations between climatic water deficit and Z_90_ and BA_90_ (*SI Appendix*, Tables S5 and S7), results of which are very similar to those of VPD (*SI Appendix*, Tables S5 and S6).

We calculate warm season (May to September) ecoregion averages of VPD and use a least-square regression analysis to model the interannual relationship between VPD and Z_90_ and BA_90_ (*SI Appendix*, Tables S8 and S9) in each ecoregion separately. Finally, we approximate the additional high-elevation forest area exposed to potential fire activity imparted through VPD trends during 1984 to 2017. This was accomplished by applying the VPD-Z_90_ regression to a linear VPD trend estimated for years 1984 and 2017. Subsequently, we find the forest area encapsulated between these two elevations in each ecoregion (*SI Appendix*, Table S8).

To determine the fraction of warm season VPD trend in each ecoregion that is directly attributable to increasing temperature, we calculate VPD based on daily minimum and daily maximum relative humidity and temperature during the warm season. We then use a decomposition analysis for attributing VPD trends which fixes relative humidity at its climatology level and allows temperature to vary. A comparison of observed VPD trends and decomposition-estimated VPD trends allows us to get a first-order estimate of the contribution of increased VPD due to warming (*SI Appendix*, Fig. S5).

All regression analyses are conducted using conventionally employed type I linear regression, assuming an independent variable (VPD) with little to no error and attributing all errors to the dependent variable (Z_90_ and BA_90_). Supplemental analysis with type II linear regression, which assumes VPD, Z_90_, and BA_90_ are dependent on an unknown parameter and allows for error in all variables, markedly enhances the proximal influence of VPD on the Z_90_ and BA_90_ (*SI Appendix*, Table S12).

Finally, climate velocity is defined as the “rate and direction” that an organism should move to maintain a constant climate, often defined using a single climate variable (50). We define the climate velocity of fire as the rate of movement of VPD, which has a strong correlation with forest fire elevation, to sustain a certain isoline. Although velocity is a three-dimensional vector, we focus exclusively on the vertical, or elevational climate velocity of VPD using (38)

$$\frac{d\left(E\right)}{d\left(t\right)}=\frac{d\left(VPD\right)/d\left(t\right)}{d\left(VPD\right)/d\left(E\right)},$$ [3]

in which $\frac{d\left(E\right)}{d\left(t\right)}$ represents elevational velocity (meters per year), $\frac{d\left(VPD\right)}{d\left(t\right)}$ signifies temporal gradient of VPD, and $\frac{d\left(VPD\right)}{d\left(E\right)}$ is elevational gradient of VPD. For each grid in each ecoregion, we calculate the average May to September value of VPD for each year, which provides $\frac{d\left(VPD\right)}{d\left(t\right)}$ through the slope of linear regression. Similarly, for each grid, we estimate the elevation and long-term average of May to September mean value, which collectively provide $\frac{d\left(VPD\right)}{d\left(E\right)}$ through linear regression analysis. Absolute changes of VPD isoline are readily calculated by multiplying the climate velocity of VPD (meters per year) by the length of record (34 y).

A detailed description of quantile regression and Mann–Kendall nonparametric trend test can be found in Koenker and Hallock (51) and Hamed and Rao (52), respectively. Furthermore, details of distribution tests including the two-sample Cramér–von Mises test, Anderson–Darling test, and Kolmogorov–Smirnov test can be found in Darling (53).

## Supplementary Material

Supplementary File

## Data Availability

All data are publicly available. Forest cover is available from the Landfire Program (https://www.landfire.gov/esp.php). Annual burned area is available from the MTBSP (https://mtbs.gov/direct-download). Level III Omernik ecoregions are available from the Environmental Protection Agency (https://www.epa.gov/eco-research/level-iii-and-iv-ecoregions-continental-united-states). Source of ignition is available from the FPA-FOD (https://www.fs.usda.gov/rds/archive/catalog/RDS-2013-0009.4). DEM is available from the National Elevation Dataset (https://apps.nationalmap.gov/downloader/#/). AVHRR-based NDVI is available at https://www.ncdc.noaa.gov/cdr/terrestrial/normalized-difference-vegetation-index. All other study data are included in the article and/or *SI Appendix*.
