# Supplementary material for: Warming enabled upslope advance in western US forest fires
Source: Proc Natl Acad Sci U S A. 2021 May 24;118(22):e2009717118. doi: 10.1073/pnas.2009717118 (PMC8179236; doi:10.1073/pnas.2009717118)
Supplement: Supplementary File [file pnas.2009717118.sapp.pdf]

Supplementary Information

for

Warming Enabled Upslope Advance in Western U.S. Forest Fires

Mohammad Reza Alizadeh[1], John T. Abatzoglou[2], Charles H. Luce[3], Jan Adamowski[1], Arvin Farid[4], Mojtaba Sadegh[4,*]

[1] Department of Bioresource Engineering, McGill University, Quebec, CANADA.
[2] Management of Complex Systems Department, University of California, Merced, Merced, CA, UNITED STATES.
[3] US Forest Service Aquatic Science Laboratory, Rocky Mountain Research Station, Boise, ID, UNITED STATES.
[4] Department of Civil Engineering, Boise State University, Boise, ID, UNITED STATES.
[*]Correspondence to mojtabasadegh@boisestate.edu

Throughout this document, we refer to "$\mathbf{Z_{90}}$" as the 90th percentile of normalized annual elevational distribution of burned forest in each ecoregion. Here, the term "normalized" essentially refers to the fraction of burned forest as a function of elevation. Further, we refer to "$\mathbf{BA_{90}}$" as the annual forest burned area above the 90th percentile of the forest elevational distribution in each ecoregion. Also, VPD and CWD represent vapor pressure deficit and climatic water deficit, respectively, and NDVI represents normalized difference vegetation index.

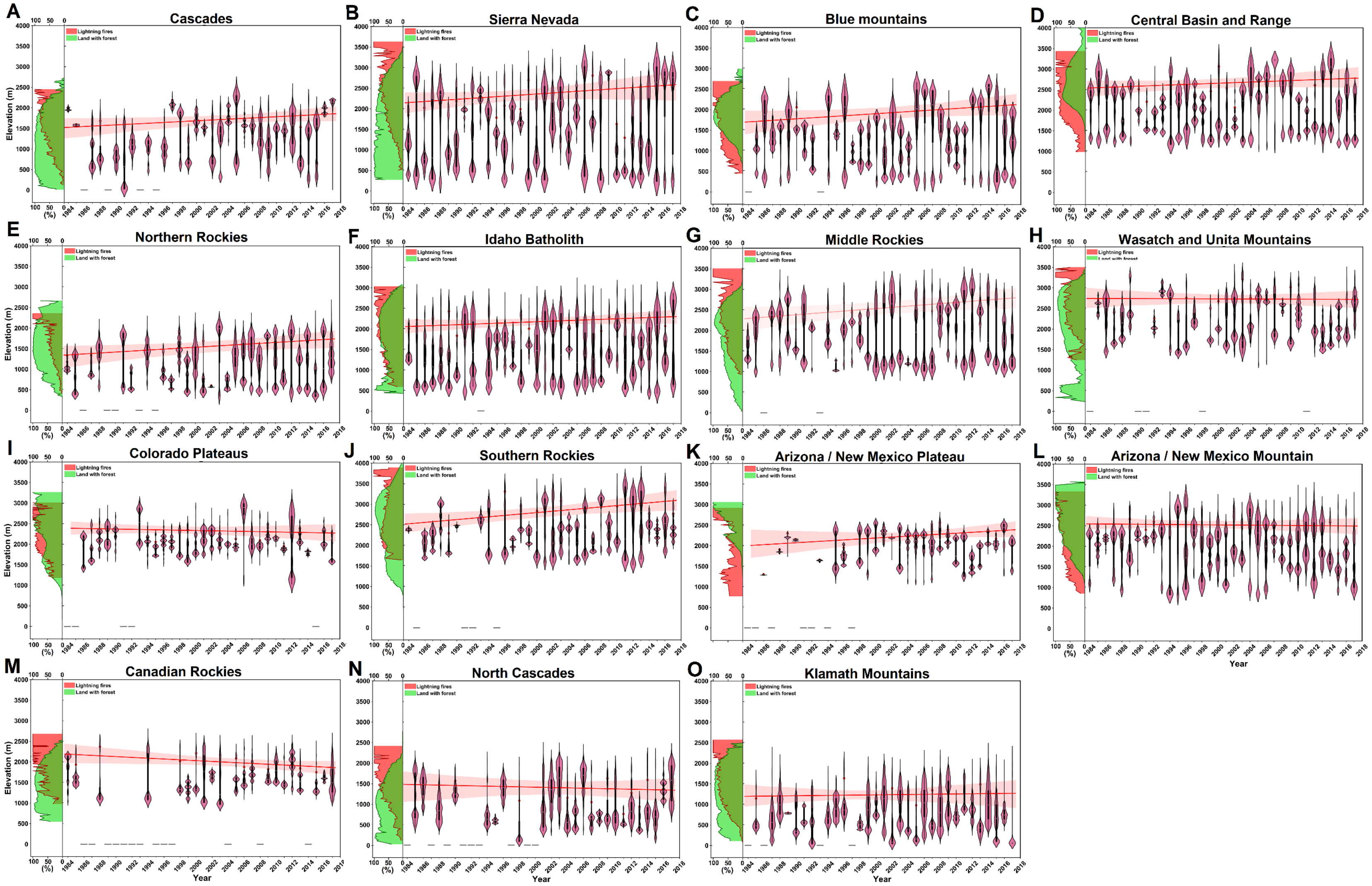

Figure S1. Time series of normalized annual elevational distribution of burned forest across ecoregions. The left panel shows the percent forest cover across elevation (green) and percent of fires that are lightning-caused (red). Violin plots for each year show normalized elevational distribution of burned forest. "Normalized" refers to the fraction of forest that is burned by elevation. Red line indicates slope of Z_{90} during 1984-2017, with uncertainty range shown with shaded area. Analysis is based on observed burned forest data.

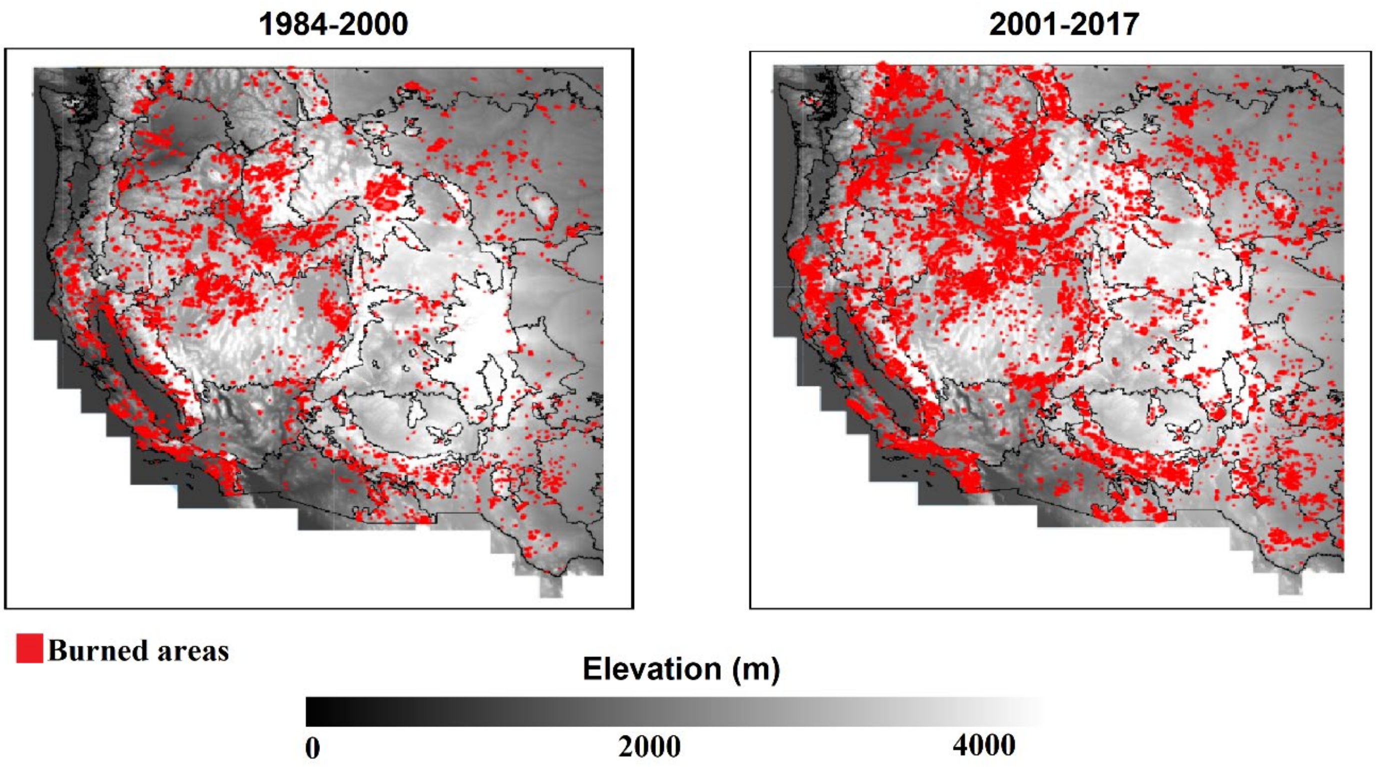

Figure S2. Spatial distribution of burned forest in the periods of 1984-2000 and 2001-2017 (marked with red) on a digital elevation map (grey scale). Visualization is based on observed burned forest data.

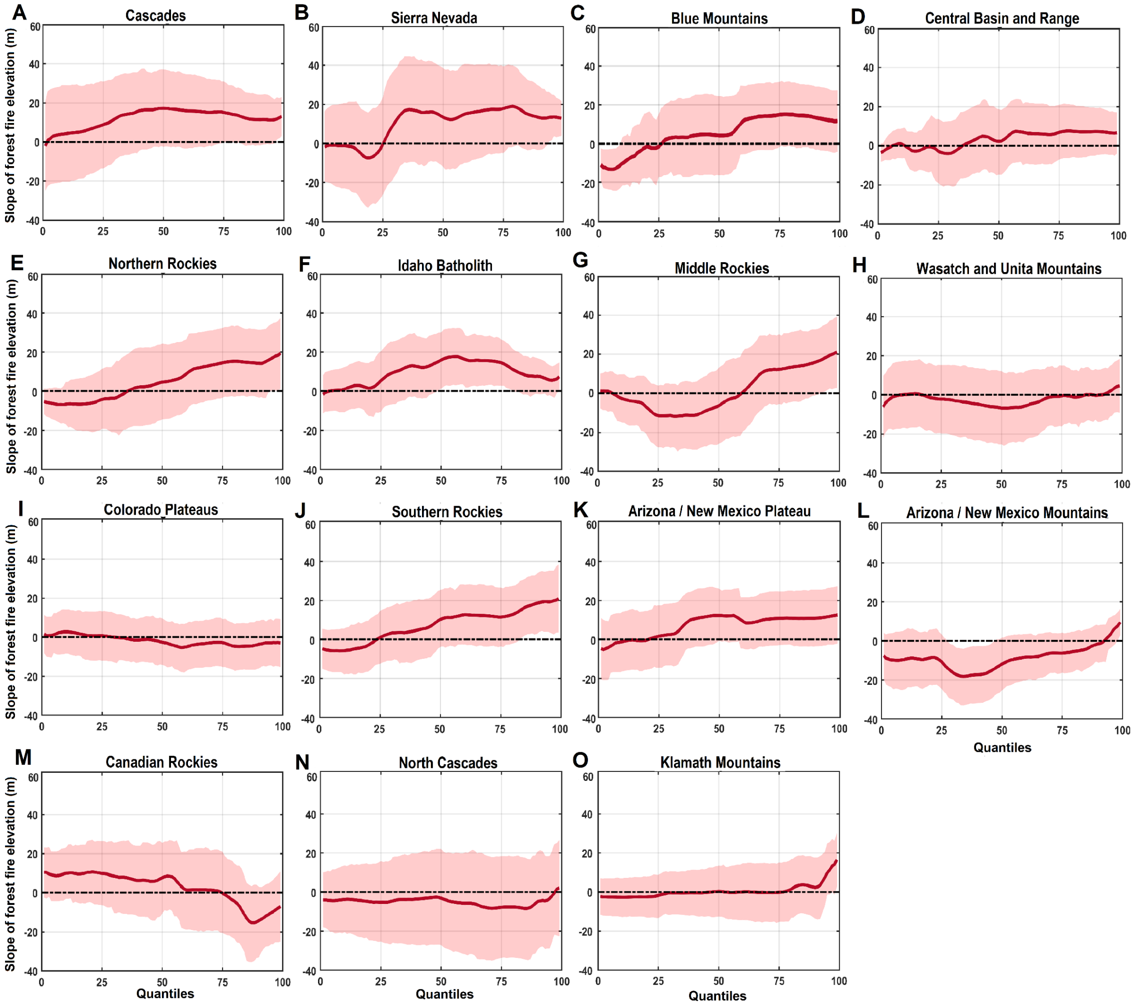

Figure S3. Quantile regression analysis of normalized annual elevations of burned forest. A: Cascades, **B:** Sierra Nevada, **C:** Blue Mountains, **D:** Central Basin and Range, **E:** Northern Rockies, **F:** Idaho Batholith, **G:** Middle Rockies, **H:** Wasatch and Unita Mountains, **I:** Colorado Plateaus, **J:** Southern Rockies, **K:** Arizona / New Mexico Plateau, **L:** Arizona / New Mexico Mountains, **M:** Canadian Rockies, **N:** North Cascades, **O:** Klamath Mountains / California High North Coast Range. Quantile regression is conducted on normalized annual elevational distribution of burned forest in each ecoregion to quantify elevational trends in burned forest across different elevations. Smaller quantiles represent lower-elevation fires and larger quantiles represent higher-elevation fires. Trends in the 90th quantile is equivalent to Z_{90} as explained in the main paper.

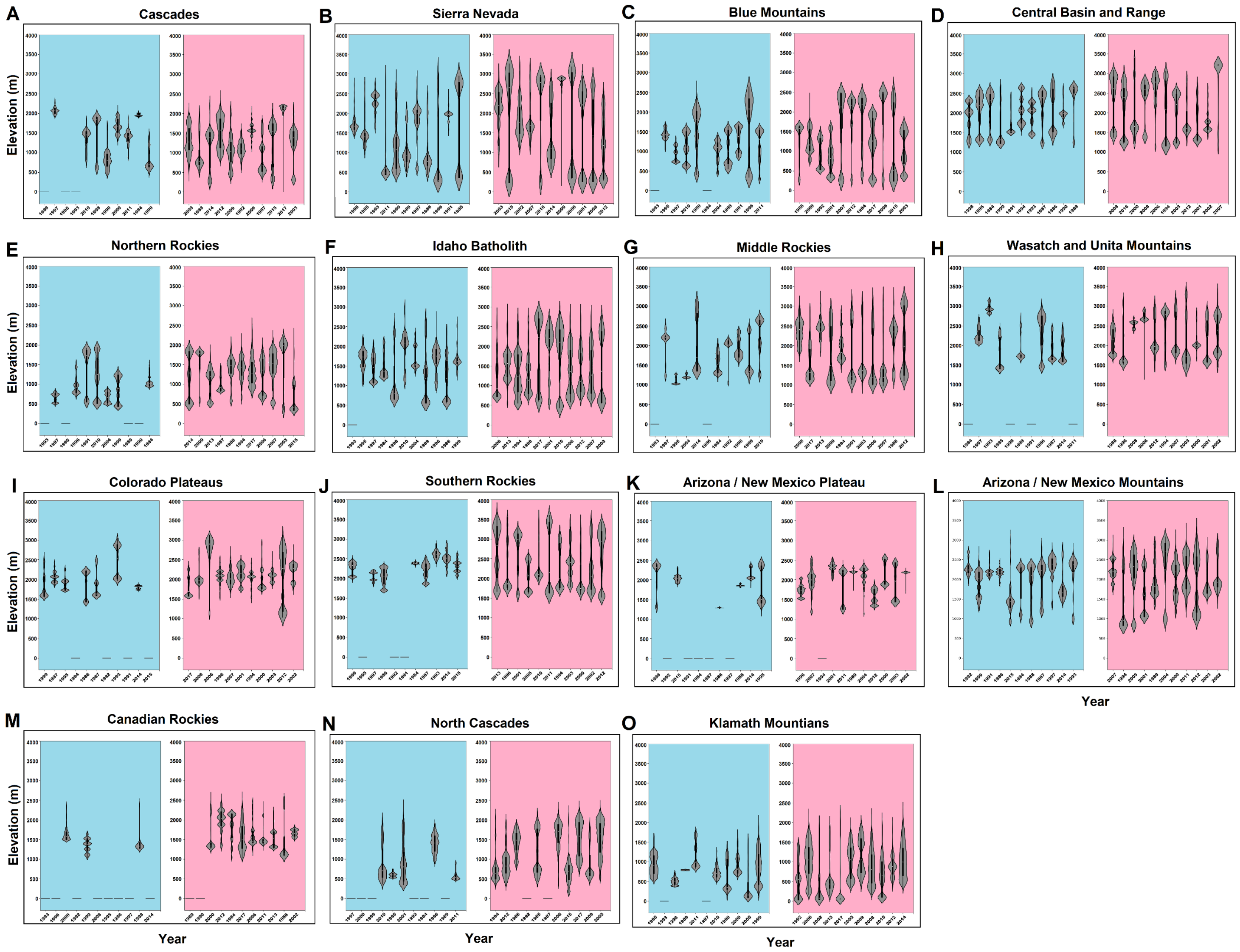

Figure S4. Normalized annual elevational distribution of burned forest for low-VPD warm seasons (blue background; lower tercile of VPD) and high-VPD warm seasons (red background; higher tercile of VPD). No-fire years are marked with dashes on a zero elevation. Warm season is defined as May-September. Ecoregion-average warm-season VPD is used. Visualization is based on observed burned forest data.

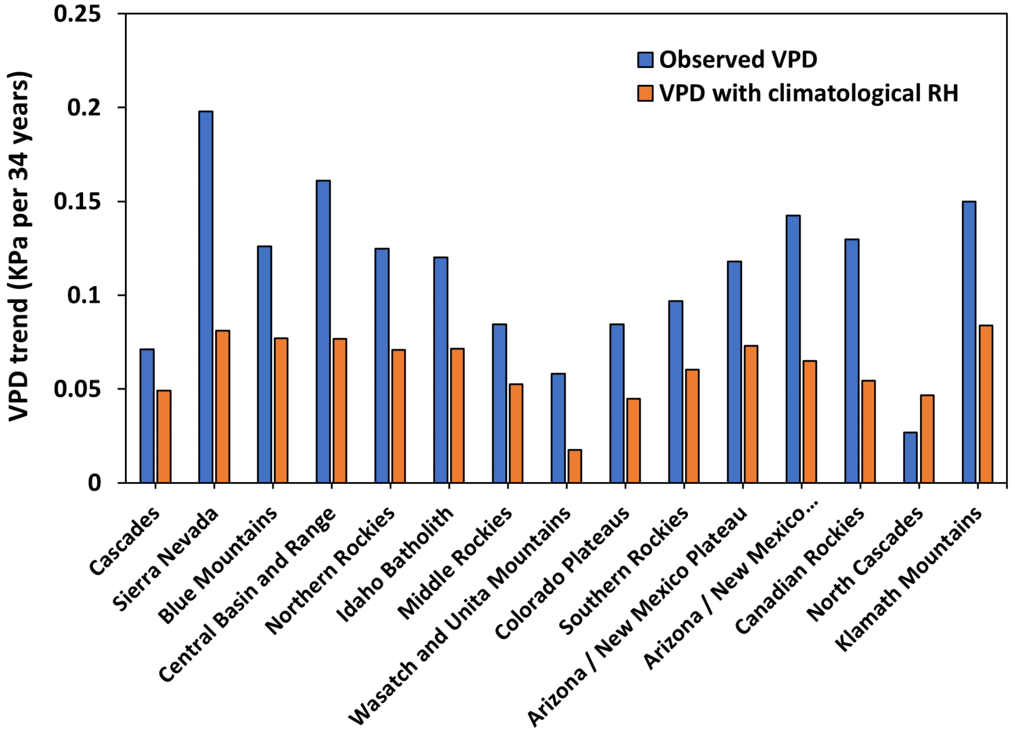

Figure S5. Observed warm-season VPD trends (blue) and trends attributed to warming (orange) during 1984-2017 for each ecoregion. To decompose the VPD trends, relative humidity was prescribed at mean values for 1984-2017 for each ecoregion allowing for an estimate of VPD trends exclusively tied to changes in air temperature. Across all ecoregions, a median of 60% of VPD trends can be attributed to changes in air temperature.

Table S1. Trends in Z_{90} and warm-season VPD isoline elevations in each ecoregion during 1984-2017, as well as their 95% confidence interval. Linear trend analysis is conducted on the observed burned forest elevation and May-September ecoregion-average values of observed VPD. Trend in VPD elevation is calculated as a product of temporal gradient of VPD and elevational gradient of VPD.

Ecoregion	Trends (m) in Z_{90} in during 1984-2017*			Trends (m) in elevation of VPD isolines during 1984-2017		
	Average (Fig. 1)	Lower bound	Upper bound	Average (Fig. 4)	Lower bound	Upper bound
Cascades	338.60	-50.92	728.13	601.83	23.22	1331.30
Sierra Nevada	443.87	-107.00	994.74	514.47	198.86	840.71
Blue Mountains	420.03	-97.95	938.01	483.20	97.06	892.11
Central Basin and Range	243.65	-168.70	656.01	287.54	29.53	555.58
Northern Rockies	404.96	-94.89	904.80	284.99	13.67	568.32
Idaho Batholith	252.81	-48.64	554.25	441.89	99.83	798.40
Middle Rockies	505.73	-33.69	1045.15	295.02	151.32	757.45
Wasatch and Unita Mountains	-16.45	-400.64	367.74	170.65	73.13	429.83
Colorado Plateaus	-110.11	-463.11	242.89	182.26	27.08	404.78
Southern Rockies	549.65	85.63	1013.66	358.39	32.52	704.44
Arizona / New Mexico Plateau	296.45	-94.55	687.45	147.45	11.97	289.07
Arizona / New Mexico Mountains	-52.60	-376.86	271.66	191.08	2.14	383.30
Canadian Rockies	-315.08	-693.39	63.23	590.93	59.32	1218.45
North Cascades	-109.38	-736.04	517.29	149.47	62.66	375.68
Klamath Mountains / …	57.49	-411.87	526.86	474.03	71.57	916.05
Median: all ecoregions	252.81	-107	656.01	295.02	59.32	704.44

* The slope of the 90th quantile (m/yr) of normalized annual elevational distribution of burned forest is multiplied by the number of years with forest fire activity to calculate the increase in Z_{90} for each ecoregion during 1984-2017. Similarly, the lower and upper bounds of the 95% confidence interval, CI, for the slope of the 90th quantile (m/yr) are multiplied by the number of years with forest fire activity to estimate the 95% CI of Z_{90} trends for each ecoregion. A similar approach was adopted for calculating uncertainty ranges of trends in elevation of VPD isolines.

Table S2. Average forest burned area (BA; ha/yr) in different elevation bands during 1984-2000 and 2001-2017. Annual observed burned forest data is used in this analysis.

Ecoregion	Periods	Elevation bands (m)						
		0- 500	500-1000	1000-1500	1500-2000	2000-2500	2500-3000	>3000
Cascades	BA (ha/yr): 1984-2000	679.78	2838.30	2827.46	397.01	125.47	0	0
	BA (ha/yr): 2001-2017	2777.41	3511.62	5328.51	4173.41	1691.41	0.66	0
	Percent change	308.57	23.72	88.46	951.20	1248.09	Infinity	–
Sierra Nevada	BA (ha/yr): 1984-2000	6075.86	3641.87	2138.59	1621.43	2351.35	1168.20	433.27
	BA (ha/yr): 2001-2017	11516.02	3690.85	4690.40	4812.67	4577.02	7587.13	4130.01
	Percent change	89.54	1.34	119.32	196.82	94.66	549.47	853.22
Blue Mountains	BA (ha/yr): 1984-2000	3540.48	788.74	1475.29	3936.94	8962.57	2132.39	0
	BA (ha/yr): 2001-2017	6931.75	1141.10	1673.02	3467.15	12131.10	7903.48	0
	Percent change	95.79	44.67	13.40	-11.93	35.35	270.64	–
Central Basin and Range	BA (ha/yr): 1984-2000	0	0	3552.39	2500.95	1538.31	2014.10	2389.94
	BA (ha/yr): 2001-2017	0	0	3488.21	3619.49	3364.19	5160.87	7452.70
	Percent change	–	–	-1.81	44.72	118.69	156.24	211.84
Northern Rockies	BA (ha/yr): 1984-2000	57.69	1089.51	2852.94	3618.33	130.23	0	0
	BA (ha/yr): 2001-2017	16570.20	6025.08	9294.92	5981.86	2394.39	110.22	0
	Percent change	28620.47	453.01	225.80	65.32	1738.56	Infinity	–
Idaho Batholith	BA (ha/yr): 1984-2000	523.11	14076.26	7699.96	10626.47	3372.21	1209.40	0
	BA (ha/yr): 2001-2017	9863.45	22011.07	12523.41	14291.34	9897.37	4390.95	21.88
	Percent change	1785.55	56.37	62.64	34.49	193.50	263.07	Infinity
Middle Rockies	BA (ha/yr): 1984-2000	0	2808.79	9869.70	2535.82	17576.38	8943.37	2672.64
	BA (ha/yr): 2001-2017	0	3929.46	14460.50	2511.14	4516.76	5045.50	2617.21
	Percent change	–	39.90	46.51	-0.97	-74.30	-43.58	-2.07
Wasatch and Unita Mountains	BA (ha/yr): 1984-2000	0	0	328.46	700.79	595.03	462.56	248.13
	BA (ha/yr): 2001-2017	0	0	604.58	3500.81	1413.64	2901.13	616.10
	Percent change	–	–	84.07	399.55	137.58	527.19	148.29
Colorado Plateaus	BA (ha/yr): 1984-2000	0	0	40.17	1290.25	1593.77	185.43	0.00
	BA (ha/yr): 2001-2017	0	0	5244.04	1923.38	4161.00	1559.95	1099.09
	Percent change	–	–	12953.32	49.07	161.08	741.24	Infinity
Southern Rockies	BA (ha/yr): 1984-2000	0	0	0	3538.27	765.98	1119.00	1495.05
	BA (ha/yr): 2001-2017	0	0	0	9361.08	7214.71	3763.06	11445.11
	Percent change	–	–	–	164.57	841.90	236.29	665.53
Arizona / New Mexico Plateau	BA (ha/yr): 1984-2000	0	0	223.00	245.15	596.30	208.78	0
	BA (ha/yr): 2001-2017	0	0	295.22	655.90	1472.37	235.62	0
	Percent change	–	–	32.38	167.54	146.92	12.86	–
Arizona / New Mexico Mountains	BA (ha/yr): 1984-2000	0	5383.76	1823.16	2628.46	5258.06	2422.62	3196.24
	BA (ha/yr): 2001-2017	0	10949.32	11754.11	25497.89	15585.62	15346.07	1182.18
	Percent change	–	103.38	544.71	870.07	196.41	533.45	-63.01
Canadian Rockies	BA (ha/yr): 1984-2000	0	0	10879.43	955.73	2416.84	1141.70	0
	BA (ha/yr): 2001-2017	0	45892.90	6662.99	8429.86	4490.71	30.31	0
	Percent change	–	Infinity	-38.76	782.04	85.81	-97.35	–
North Cascades	BA (ha/yr): 1984-2000	2467.16	8277.75	3146.34	604.62	40.04	0	0
	BA (ha/yr): 2001-2017	4184.46	8216.20	4643.48	6657.26	2675.91	0	0
	Percent change	69.61	-0.74	47.58	1001.07	6582.42	–	–
Klamath Mountains / …	BA (ha/yr): 1984-2000	8675.28	10749.48	3274.17	1050.98	309.97	0	0
	BA (ha/yr): 2001-2017	18632.03	13063.33	8246.85	3897.69	251.22	0	0
	Percent change	114.77	21.53	151.88	270.86	-18.95	–	–
Median: all ecoregions	Percent change	114.77	42.29	73.35	167.54	146.92	270.64	438.69

Table S3. Statistical difference tests between slopes of quantile regression for the upper half (50%-100%) versus the lower half (0%-49%) of burned forest elevations. Test results point to statistically significant difference between upslope trends in higher-elevation fires (50 to 100 quantiles) and in lower-elevation fires (0 to 49 quantiles) for all studied ecoregions. When combined with Fig. S1, these results point to statistically significant larger trends in upslope advancement of higher-elevation fires as compared to lower-elevation fires.

Ecoregion	Anderson-Darling test		Cramer Von Mises test		Kolmogorov-Smirnov test		Kruskal-Wallis test		Two-sample t-test		Mann-Whitney U-test	
	Test result	P-value	Test result	P-value	Test result	P-value	Test result	P-value	Test result	P-value	Test result	P-value
All Ecoregions	1	≤0.0005	1	1	1	≤1.76E-22	1	≤7.97E-18	1	≤1.57E-17	1	≤8.21E-18

Table S4. Interannual Pearson correlation analysis between May-September cloud-to-ground lightning strikes and Z_{90} and BA_{90}, as well as May-September VPD during 1990-2017. Total lightning strikes in each ecoregion and ecoregion-average VPD, as well as observed burned forest data is used in this analysis.

Ecoregion code	Ecoregion	Lightning – Z_{90}		Lightning – Annual BA_{90}		Lightning – VPD	
		Correlation coefficient	P-value	Correlation coefficient	P-value	Correlation coefficient	P-value
4	Cascades	-0.10	0.61	0.27	0.21	0.34	0.08
5	Sierra Nevada	0.23	0.25	0.02	0.93	0.00	1.00
11	Blue Mountains	-0.09	0.66	0.06	0.77	0.15	0.44
13	Central Basin and Range	0.25	0.21	0.01	0.96	-0.06	0.77
15	Northern Rockies	-0.08	0.71	-0.18	0.40	0.08	0.68
16	Idaho Batholith	0.10	0.63	-0.41	0.04	-0.25	0.21
17	Middle Rockies	0.00	0.98	-0.19	0.34	-0.25	0.20
19	Wasatch and Unita Mountains	0.26	0.22	0.04	0.84	0.06	0.78
20	Colorado Plateaus	-0.07	0.75	-0.28	0.19	-0.09	0.66
21	Southern Rockies	-0.23	0.27	-0.32	0.13	0.13	0.50
22	Arizona / New Mexico Plateau	0.25	0.24	0.13	0.55	0.11	0.57
23	Arizona / New Mexico Mountains	-0.20	0.31	0.07	0.72	0.13	0.51
41	Canadian Rockies	-0.13	0.60	-0.27	0.28	-0.08	0.67
77	North Cascades	-0.16	0.48	-0.29	0.27	0.21	0.28
78	Klamath Mountains	0.16	0.44	-0.07	0.75	0.41	0.03

Table S5. Interannual Pearson correlation analysis between Z_{90} and May-September VPD and May-September CWD. Ecoregion-average VPD and CWD, as well as observed burned forest data is used in this analysis. Analyses are presented with original data as well as their linearly detrended counterparts. Analysis with detrended data helps demonstrate that the relationship between the two variables is not merely due to long-term increasing trends in both variables, and the two variables are indeed correlated regardless of their trends.

Ecoregion code	Ecoregion	Z_{90} – VPD		Detrended Z_{90} – Detrended VPD		Z_{90} – CWD		Detrended Z_{90} – Detrended CWD	
		Correlation coefficient	P-value	Correlation coefficient	P-value	Correlation coefficient	P-value	Correlation coefficient	P-value
4	Cascades	-0.08	0.69	-0.17	0.37	-0.10	0.59	-0.19	0.32
5	Sierra Nevada	0.51	0.00	0.44	0.01	0.45	0.01	0.41	0.02
11	Blue Mountains	0.42	0.02	0.35	0.05	0.41	0.02	0.38	0.03
13	Central Basin and Range	0.33	0.06	0.27	0.12	0.24	0.18	0.21	0.24
15	Northern Rockies	0.32	0.09	0.25	0.19	0.33	0.08	0.26	0.17
16	Idaho Batholith	0.28	0.12	0.18	0.31	0.21	0.25	0.15	0.41
17	Middle Rockies	0.49	0.00	0.45	0.01	0.43	0.01	0.41	0.02
19	Wasatch and Unita Mountains	0.42	0.02	0.43	0.02	0.38	0.04	0.38	0.04
20	Colorado Plateaus	0.16	0.41	0.19	0.32	0.24	0.21	0.24	0.22
21	Southern Rockies	0.65	0.00	0.61	0.00	0.57	0.00	0.53	0.00
22	Arizona / New Mexico Plateau	0.21	0.29	0.19	0.34	0.26	0.18	0.30	0.13
23	Arizona / New Mexico Mountains	0.41	0.02	0.46	0.01	0.46	0.01	0.49	0.00
41	Canadian Rockies	0.24	0.30	0.10	0.67	0.34	0.14	0.44	0.04
77	North Cascades	0.35	0.09	0.37	0.07	0.41	0.04	0.43	0.03
78	Klamath Mountains	0.25	0.18	0.25	0.18	0.19	0.32	0.18	0.33

Table S6. Interannual Pearson correlation analysis between May-September VPD and forest burned area above the thresholds of 2,000 m (BA_{2000}), 2,500 m (BA_{2500}) and the 90th percentile of forest elevational distribution in each ecoregion (BA_{90}). Ecoregion-average VPD and observed burned forest data is used in this analysis.

Ecoregion code	Ecoregion	BA_{2000} – VPD		BA_{2500} – VPD		BA_{90} – VPD	
		Correlation coefficient	P-value	Correlation coefficient	P-value	Correlation coefficient	P-value
4	Cascades	0.63	0.00	0.34	0.20	0.70	0.00
5	Sierra Nevada	0.59	0.00	0.53	0.00	0.57	0.00
11	Blue Mountains	0.31	0.10	0.33	0.22	0.36	0.04
13	Central Basin and Range	0.51	0.00	0.43	0.01	0.41	0.02
15	Northern Rockies	0.60	0.00	0.49	0.10	0.56	0.00
16	Idaho Batholith	0.56	0.00	0.63	0.00	0.58	0.00
17	Middle Rockies	0.50	0.01	0.49	0.01	0.56	0.00
19	Wasatch and Unita Mountains	0.58	0.00	0.46	0.01	0.57	0.00
20	Colorado Plateaus	0.65	0.00	0.64	0.00	0.63	0.00
21	Southern Rockies	0.64	0.00	0.61	0.00	0.64	0.00
22	Arizona / New Mexico Plateau	0.19	0.35	0.20	0.36	0.40	0.04
23	Arizona / New Mexico Mountains	0.63	0.00	0.61	0.00	0.58	0.00
41	Canadian Rockies	0.24	0.77	0.03	0.91	0.06	0.80
77	North Cascades	0.57	0.02	0.62	0.03	0.54	0.02
78	Klamath Mountains	0.50	0.02	0.09	0.75	0.51	0.01

Table S7. Interannual Pearson correlation analysis between May-September CWD and forest burned area above the thresholds of 2,000 m (BA_{2000}), 2,500 m (BA_{2500}) and the 90th percentile of forest elevational distribution in each ecoregion (BA_{90}). Ecoregion-average CWD and observed burned forest data is used in this analysis.

Ecoregion code	Ecoregion	BA_{2000} – CWD		BA_{2500} – CWD		BA_{90} – CWD	
		Correlation coefficient	P-value	Correlation coefficient	P-value	Correlation coefficient	P-value
4	Cascades	0.56	0.00	0.09	0.73	0.59	0.00
5	Sierra Nevada	0.56	0.00	0.48	0.00	0.58	0.00
11	Blue Mountains	0.36	0.05	0.37	0.16	0.44	0.01
13	Central Basin and Range	0.39	0.02	0.29	0.10	0.33	0.05
15	Northern Rockies	0.70	0.00	0.44	0.15	0.65	0.00
16	Idaho Batholith	0.55	0.00	0.55	0.00	0.57	0.00
17	Middle Rockies	0.51	0.00	0.50	0.01	0.56	0.00
19	Wasatch and Unita Mountains	0.50	0.01	0.41	0.03	0.46	0.01
20	Colorado Plateaus	0.55	0.00	0.52	0.01	0.53	0.00
21	Southern Rockies	0.61	0.00	0.57	0.00	0.60	0.00
22	Arizona / New Mexico Plateau	0.12	0.55	0.15	0.49	0.31	0.12
23	Arizona / New Mexico Mountains	0.51	0.00	0.52	0.00	0.46	0.01
41	Canadian Rockies	0.60	0.00	0.57	0.01	0.72	0.00
77	North Cascades	0.67	0.00	0.69	0.01	0.65	0.00
78	Klamath Mountains	0.54	0.01	-0.03	0.92	0.63	0.00

Table S8. VPD-driven trend in Z_{90} during 1984-2017, and the forest area encapsulated between VPD-estimated Z_{90} at 1984 and Z_{90} at 2017. Area of forest cover in each ecoregion and fraction of forest cover encapsulated between VPD-estimated Z_{90} at 1984 and Z_{90} at 2017 are also provided. The latter is hypothesized to be the forested land that has become vulnerable to fire due to increasing VPD trends. VPD-driven trends in Z_{90} during 1984-2017 are calculated as the product of linear VPD trend and the VPD-Z_{90} regression. Regression coefficient in terms of change in Z_{90} (m) in response to unit increase in VPD z-score (one standard deviation departure from normal; $\frac{\Delta Z_{90}\ (\mathrm{m})}{\Delta \mathrm{VPD}\ (\mathrm{z-score})}$) is also provided for each ecoregion. All analyses are based on type I linear regression analysis. VPD is used in terms of z-score to be comparable across ecoregions in terms of departure from normal conditions.

Ecoregion code	Ecoregion	VPD (z-score)		VPD-estimated Z_{90} (m)		Z_{90}-VPD relationship	Area of forest cover between Z_{90} at 1984 & Z_{90} at 2017 (km^2)	Fraction of forest cover encapsulated between Z_{90} at 1984 & Z_{90} at 2017	Area of forest cover in each ecoregion (km^2)
		1984	2017	1984	2017	Regression coefficient $\left(\frac{\Delta Z_{90}\ (\mathrm{m})}{\Delta \mathrm{VPD}\ (\mathrm{z-score})}\right)$			
4	Cascades	-0.44	0.44	1713.34	1688.17	-28.48	-698.46	-1.28%	54657.68
5	Sierra Nevada	-0.81	0.81	2165.27	2575.24	253.16	5981.51	15.61%	38329.70
11	Blue Mountains	-0.65	0.65	1780.51	2039.75	199.63	2876.87	7.70%	37384.68
13	Central Basin and Range	-0.71	0.71	2569.93	2738.94	119.71	2348.13	4.48%	52445.67
15	Northern Rockies	-0.56	0.56	1454.12	1616.40	144.84	6991.74	10.74%	65073.23
16	Idaho Batholith	-0.68	0.68	2121.02	2226.34	77.25	4186.01	9.62%	43532.04
17	Middle Rockies	-0.48	0.48	2401.16	2648.21	258.09	15719.47	18.13%	86702.96
19	Wasatch and Unita Mountains	-0.40	0.40	2664.03	2764.19	126.55	2478.38	7.75%	31967.96
20	Colorado Plateaus	-0.43	0.43	2310.42	2347.51	43.38	1346.80	3.09%	43627.82
21	Southern Rockies	-0.59	0.59	2628.52	2957.23	280.05	27973.52	27.61%	101303.67
22	Arizona / New Mexico Plateau	-0.48	0.48	2176.84	2240.49	66.93	2815.86	11.31%	24887.51
23	Arizona / New Mexico Mountains	-0.52	0.52	2459.73	2581.28	117.16	4983.27	6.89%	72321.80
41	Canadian Rockies	-0.17	0.17	1990.63	1994.93	36.59	66.01	0.42%	15656.67
77	North Cascades	-0.26	0.26	1313.88	1409.92	181.51	1606.46	6.82%	23565.23
78	Klamath Mountains	-0.60	0.60	1177.84	1298.41	99.87	2892.45	7.31%	39565.30
	Western U.S.						81568.02	11.16%	731021.9

Table S9. VPD-driven trend in annual BA_{90} during 1984-2017, calculated as the product of linear VPD trend and the VPD-BA_{90} regression. Regression coefficient in terms of change in annual BA_{90} (km^2) in response to unit increase in VPD z-score (one standard deviation departure from normal; $\frac{\Delta BA_{90}\ (km^2)}{\Delta VPD\ (z-score)}$) is provided for each ecoregion. Increase in VPD-driven annual BA_{90} (km^2) between 1984 and 2017 is also provided. All analyses are based on type I linear regression analysis. VPD is used in terms of z-score to be comparable across ecoregions in terms of departure from normal conditions.

Ecoregion code	Ecoregion	VPD (z-score)		VPD-estimated annual BA_{90} (km^2)		Annual BA_{90}-VPD relationship	Increase in VPD-driven annual BA_{90} (km^2) between 1984 & 2017 km^2 (Percent increase)
		1984	2017	1984	2017	Regression coefficient $\left(\frac{\Delta BA_{90}\ (km^2)}{\Delta VPD\ (z-score)}\right)$	
4	Cascades	-0.44	0.44	5.90	19.14	15.04	13.23 (224.30%)
5	Sierra Nevada	-0.81	0.81	13.58	41.54	17.26	27.96 (205.91%)
11	Blue Mountains	-0.65	0.65	14.37	41.66	20.99	27.29 (189.85%)
13	Central Basin and Range	-0.71	0.71	7.96	39.43	22.16	31.46 (395.05%)
15	Northern Rockies	-0.56	0.56	0.00	38.91	34.74	38.91 (-)
16	Idaho Batholith	-0.68	0.68	14.13	81.52	49.56	67.40 (477.10%)
17	Middle Rockies	-0.48	0.48	3.33	56.02	54.88	52.69 (1581.31%)
19	Wasatch and Unita Mountains	-0.40	0.40	3.60	7.62	5.03	4.02 (111.73%)
20	Colorado Plateaus	-0.43	0.43	2.32	10.41	9.41	8.09 (349.32%)
21	Southern Rockies	-0.59	0.59	2.95	28.54	21.69	25.59 (868.39%)
22	Arizona / New Mexico Plateau	-0.48	0.48	1.05	4.83	3.94	3.79 (361.23%)
23	Arizona / New Mexico Mountains	-0.52	0.52	34.81	72.30	36.05	37.50 (107.73%)
41	Canadian Rockies	-0.17	0.17	20.72	21.06	0.98	0.33 (1.60%)
77	North Cascades	-0.26	0.26	11.44	22.23	20.76	10.79 (94.38%)
78	Klamath Mountains	-0.60	0.60	13.65	48.76	29.26	35.12 (257.34%)
	Western U.S.			149.81	533.97		384.16 (256.43%)

Table S10. Interannual Pearson correlation analysis between Z_{90} and annual NDVI above the thresholds of 2,000 m ($NDVI_{2000}$) and 2,500 m ($NDVI_{2500}$) in each ecoregion. Observed burned forest data is used in this analysis. Assumption is that $NDVI_{2000}$ and $NDVI_{2500}$ represent high-elevation productivity in forests.

Ecoregion code	Ecoregion	Z_{90} – $NDVI_{2000}$		Z_{90} – $NDVI_{2500}$	
		Correlation coefficient	P-value	Correlation coefficient	P-value
4	Cascades	-0.08	0.66	-0.09	0.65
5	Sierra Nevada	-0.05	0.78	0.00	0.99
11	Blue Mountains	0.29	0.11	0.18	0.32
13	Central Basin and Range	0.03	0.87	0.01	0.94
15	Northern Rockies	0.20	0.30	-0.03	0.87
16	Idaho Batholith	-0.19	0.29	-0.18	0.32
17	Middle Rockies	0.16	0.37	0.19	0.30
19	Wasatch and Unita Mountains	0.21	0.27	0.21	0.27
20	Colorado Plateaus	-0.07	0.70	0.01	0.98
21	Southern Rockies	0.35	0.06	0.36	0.05
22	Arizona / New Mexico Plateau	0.38	0.05	0.43	0.03
23	Arizona / New Mexico Mountains	0.37	0.03	0.39	0.02
41	Canadian Rockies	0.58	0.01	0.59	0.00
77	North Cascades	0.21	0.31	0.26	0.20
78	Klamath Mountains	0.52	0.00	0.50	0.01

Table S11. Interannual Pearson correlation analysis between annual forest burned area and NDVI, both above the thresholds of 2,000 m and 2,500 m. $NDVI_{2000}/BA_{2000}$ and $NDVI_{2500}/BA_{2500}$ represent annual NDVI/area of burned forest above 2,000 m and 2,500 m, respectively. Observed burned forest data is used in this analysis. Assumption is that $NDVI_{2000}$ and $NDVI_{2500}$ represent high-elevation productivity.

Ecoregion code	Ecoregion	BA_{2000} – $NDVI_{2000}$		BA_{2500} – $NDVI_{2500}$	
		Correlation coefficient	P-value	Correlation coefficient	P-value
4	Cascades	0.18	0.37	-0.41	0.11
5	Sierra Nevada	0.10	0.59	-0.13	0.48
11	Blue Mountains	0.21	0.27	0.29	0.28
13	Central Basin and Range	0.34	0.05	0.14	0.42
15	Northern Rockies	0.11	0.64	-0.26	0.41
16	Idaho Batholith	-0.18	0.32	-0.19	0.29
17	Middle Rockies	0.17	0.39	0.19	0.32
19	Wasatch and Unita Mountains	0.28	0.14	0.17	0.37
20	Colorado Plateaus	0.29	0.13	0.34	0.08
21	Southern Rockies	0.38	0.04	0.42	0.03
22	Arizona / New Mexico Plateau	0.19	0.36	0.08	0.73
23	Arizona / New Mexico Mountains	0.35	0.04	0.30	0.09
41	Canadian Rockies	0.17	0.47	0.28	0.26
77	North Cascades	-0.06	0.83	-0.12	0.70
78	Klamath Mountains	0.33	0.12	-0.43	0.11

Table S12. Regression slopes between May-September VPD and Z_{90} and BA_{90}, based on type II linear regression analyses (complementary to Tables S8 & S9 that use type I regression analyses). Observed burned forest data is used in this analysis. VPD is used in terms of z-score to be comparable across ecoregions in terms of departure from normal conditions.

Ecoregion code	Ecoregion	Z_{90}-VPD	Annual BA_{90}-VPD
		Regression coefficient (type II) $\left(\frac{\Delta Z_{90}\ (m)}{\Delta VPD\ (z-score)}\right)$	Regression coefficient (type II) $\left(\frac{\Delta BA_{90}\ (km^2)}{\Delta VPD\ (z-score)}\right)$
4	Cascades	-372.91	21.36
5	Sierra Nevada	496.56	30.13
11	Blue Mountains	480.11	57.99
13	Central Basin and Range	363.44	54.33
15	Northern Rockies	447.10	65.73
16	Idaho Batholith	276.57	85.48
17	Middle Rockies	521.41	98.34
19	Wasatch and Unita Mountains	301.29	8.95
20	Colorado Plateaus	270.91	15.08
21	Southern Rockies	429.53	34.11
22	Arizona / New Mexico Plateau	318.76	9.89
23	Arizona / New Mexico Mountains	284.24	62.53
41	Canadian Rockies	407.89	47.55
77	North Cascades	521.18	38.05
78	Klamath Mountains	395.98	57.50
